# Bacterial production of ciprofloxacin and potential usage as a radiotracer

**DOI:** 10.1371/journal.pone.0291342

**Published:** 2023-11-09

**Authors:** Kadriye Busra Karatay, Nihal Dogruoz Gungor, Batu Colak, Fazilet Zumrut Biber Muftuler, Omer Aras

**Affiliations:** 1 Department of Nuclear Applications, Institute of Nuclear Sciences, Ege University, Izmir, Turkey; 2 Department of Biology, Faculty of Science, Istanbul University, Istanbul, Turkey; 3 Institute of Graduate Studies in Sciences, Istanbul University, Istanbul, Turkey; 4 Department of Radiology, Memorial Sloan Kettering Cancer Center, New York, New York, United States of America; Qassim University, SAUDI ARABIA

## Abstract

Infectious diseases caused by bacteria that have become resistant to antibiotics have increased in prevalence, necessitating new methods for their diagnosis and treatment. The aim of this study was to compare the efficacy of synthetic ciprofloxacin to that of organic ciprofloxacin produced by cave microorganisms, as well as to evaluate the feasibility of using organic ciprofloxacin radiolabeled with technetium-99m as an imaging agent. Organic ciprofloxacin produced by cave bacteria isolated from sediment taken from the dark zone of Antalya’s "Yark Sinkhole," (Turkey’s 14^th^ deepest cave), was purified using high-performance liquid chromatography. Purified organic ciprofloxacin and standard ciprofloxacin were radiolabeled with technetium-99m (^99m^Tc), and their uptake by pathogenic microorganisms as well as potential as an imaging agent were examined. According to thin-layer radiochromatography, radiolabeling efficiencies were 98.99 ± 0.34 (n = 7) and 91.25 ± 1.84 (n = 7) for radiolabeled organic ciprofloxacin and standard ciprofloxacin respectively. The binding efficiency of radiolabeled organic ciprofloxacin at the 240^th^ minute was higher compared with radiolabeled standard ciprofloxacin, especially with *P*.*aeruginosa*, *MRSA*, *VRE* and *E*.*coli*. The results demonstrate that radiolabeling with ^99m^Tc does not alter the biological behavior of organic ciprofloxacin, and radiolabeled organic ciprofloxacin has potential as an imaging agent for the detection of bacterial infection. The original value of the study is the monitoring of the antibiofilm effects of untouched cave-derived organic antibiotics by radiolabeling with a radionuclide.

## Introduction

With the widespread use of antibiotics worldwide, there has been a corresponding rise in antibiotic resistance, whereby bacteria become resistant to antibiotics [1]. Currently, antibiotic resistance is recognized as a major public health issue, causing approximately 700,000 deaths globally per year, and potentially causing approximately 9.5 million deaths globally per year if the current level of antibiotic resistance increases by 40% [2]. Consequently, innovative approaches to tackle the rising problem of antibiotic resistance have been proposed, with one such approach entailing the use of organic antibiotics from cave microorganisms as an alternative to today’s synthetic antibiotics. Indeed, various extreme habitats host microorganisms that have not been discovered or evaluated for antimicrobial activity [3, 4].

Moreover, there is a need for innovative imaging techniques that can be used for the diagnosis and treatment of bacterial infection. While many imaging techniques including computed tomography or magnetic resonance imaging can identify anatomical changes and discern inflammatory processes, they cannot easily discern bacterial infections from non-bacterial infections. Nuclear medicine techniques may offer better solutions, and many efforts have been made to develop radiopharmaceuticals that can distinguish between bacterial infection and sterile inflammation. In this context, pre-clinical studies have evaluated the use of several antimicrobial peptides, antibiotics, antibiotic peptides, and chemotactic peptides, radiolabeled with different radionuclides, e.g., ^67^Gallium, technetium-99m (^99m^Tc), ^111^Indium, ^18^Fluorine, and ^131^Iodine [5–7].

The main hypothesis of our study is that organic antibiotic ciprofloxacin (o-CIP), produced by cave microorganisms from the dark zone of Antalya’s " Yarık Sinkhole," Turkey’s 14^th^ deepest cave (2023), can be radiolabeled with technetium-99m (^99m^Tc) and thereby be used as a radiotracer for the diagnosis and treatment of bacterial infection. Specifically, o-CIP produced by the *Micrococcus luteus* bacteria from the “Yarık Sinkhole” was isolated and purified. The purified o-CIP and its synthetic counterpart, s-CIP (CAS No.:85721-33-1, Merck) were applied to several pathogenic microorganisms to determine their antimicrobial activity. Then, o-CIP and s-CIP were both radiolabeled with technetium-99m (^99m^Tc) and their radiolabeling efficiencies (^99m^Tc-o-CIP) were determined. Of note, ^99m^Tc is a frequently used radionuclide in nuclear medicine imaging. Stability and lipophilicity studies concerning ^99m^Tc-o-CIP and ^99m^Tc-s-CIP were carried out. Lastly, the binding efficiencies of ^99m^Tc-o-CIP and ^99m^Tc-s-CIP with different pathogenic microorganisms were examined to ascertain their potential as radiotracers for imaging bacterial infection. It was observed that both o-CIP and s-CIP were radiolabeled successfully, and both ^99m^Tc-o-CIP and ^99m^Tc-s-CIP were efficiently incorporated by pathogenic microorganisms. Thus, both ^99m^Tc-o-CIP and ^99m^Tc-s-CIP may play an important role in the diagnosis and treatment of infectious diseases caused by bacteria.

## Materials and methods

### Sample collection

**Fig 1:**
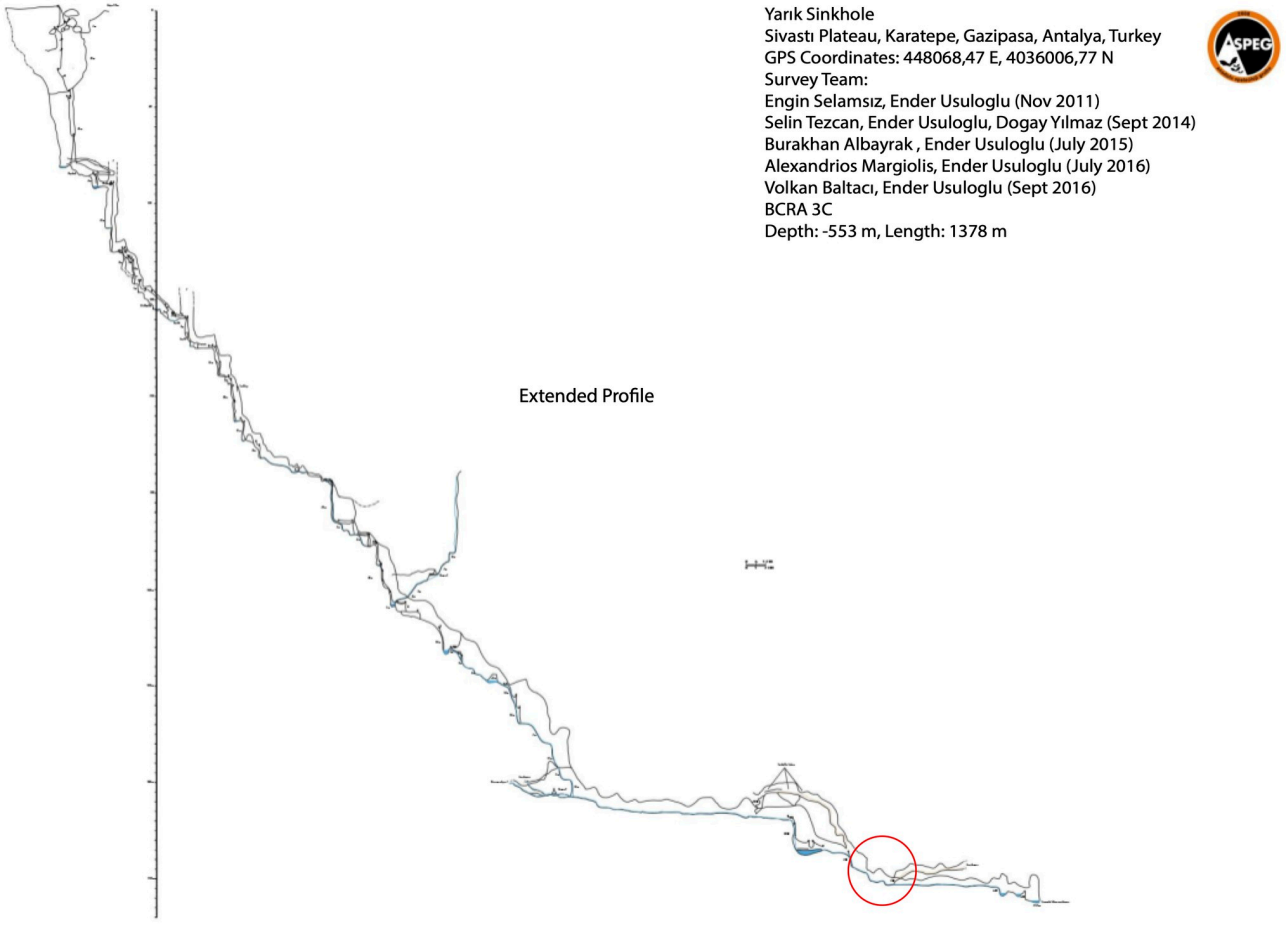
Extended profile of the Yarık Sinkhole.

Bacteria were isolated from the surface of a sample taken from 80 meters deep in the “Yarık Sinkhole” (Fig 1).

When isolating the bacteria from the sample, a transparent zone was observed around the colony in the petri dish, which aided in the isolation of pure culture. The isolated pure culture was given the code Y154 and its genomic DNA was extracted using a bacterial DNA isolation kit (Thermo Fisher Scientific, BM Labosis, Ankara, Turkey). Extracted bacterial DNA was amplified using 27F (5′‐AGAGTTTGATCCTGGCTCAG‐3′) and 1492R (5′‐GGTTACCTTGTTACGACTT‐3′) universal primers. A 50 μL mixture comprising 20 nM 27F primer, 20 nM 1492R primer, 10 ng cDNA, and 2.5 U Taq DNA polymerase (Bioline, UK) was reacted in a single block thermal cycler (Bio-rad, California, USA). Cycle conditions were 95°C for 1 min initial DNA denaturation, followed by 35 cycles consisting of denaturation for 15 s at 95°C, annealing for 15 s at 55°C, and extension for 10 s at 72°C. The polymerase chain reaction (PCR) product was sequenced using the Sanger sorting method. The sequence was read using the ABI 3130 sequencer (Applied Biosystems, BM Labosis, Ankara, Turkey). After sequence analysis, in comparison with ABI 3130 library results, we determined our bacteria to be " *Micrococcus luteus*".

The McFarland Standards were used to determine the number of cultivated bacteria in liquid medium (sterile phosphate buffer solution (PBS), sterile isotonic 0.9% NaCl solution, etc.). Specifically, cultivated bacteria (in 1/2 Mueller Hinton Agar (MHA)) were transferred to a certain amount of liquid medium and after turbidity occurred, absorbance values at 620 nm were read using a spectrophotometer housed at Ege University Institute of Nuclear Sciences. An absorbance value of 0.1 corresponded to a McFarland value of 0.5 (10^8^ bacteria / mL). The number of bacteria in 1 mL was determined and subsequent experiments were carried out using this standard amount.

### Liquid chromatography quadrupole time-of-flight mass spectrometry (LC/QTOF/MS) and high-performance liquid chromatography (HPLC) analysis

#### LC/QTOF/MS analysis

LC/Q-TOF/MS analysis was carried out at the Ege University Central Research Test and Analysis Laboratory Application and Research Center to detect antibiotics in the species of bacteria isolated from the “Yarık Sinkhole". MS analysis was carried out using an Agilent 6550 iFunnel QTOF-MS system (Agilent Technologies, Santa Clara, USA) with Dual Agilent Jet Stream electrospray ionization, desiccant gas flow 14.0 L/min, nebulizer gas pressure 35 psi, drying gas temperature 290 °C, sheath gas temperature 400 °C, and sheath nitrogen gas flow 12 L/min. Mass spectra were recorded in the negative ionization mode in the mass range of 50–1800 m/z.

#### HPLC purification and analysis

According to the ABI 3130 library results, the species of bacteria isolated from the “Yarik Sinkhole” was determined to be *Micrococcus luteus*. A low-pressure gradient HPLC system (LC-10ATvp quaternary pump, SPD-M20A DAD detector, CTO-10AS column oven, SIL 20A-HT auto sampler, FRC-10A fraction collector, and 5-μm C18-ODS column (I.D. 250 × 4.6 mm); GL Sciences, Tokyo, Japan) was used to purify and analyze o-CIP produced by *Micrococcus luteus* isolated from the cave environment [8]. Acetonitrile and phosphate buffer solution (pH 7) (18:82 v/v) were used as the mobile phase. o-CIP was eluted at a rate of 1 mL/min. UV detections were achieved at 316 nm. The HPLC chromatogram of o-CIP was evaluated and HPLC peaks were ascertained using the fraction collector in the HPLC system. Of note, the calibration curve for HPLC analysis of o-CIP was generated based on s-CIP. In addition to HPLC analysis of o-CIP, HPLC analysis of s-CIP was also performed under the same conditions to compare the two types of ciprofloxacin. HPLC analysis was repeated 7 times to ensure the accuracy of the results. After that, o-CIP was lyophilized.

### Radiolabeling and quality control studies of o-CIP and s-CIP

^99m^Tc in the form of Na^99m^TcO_4_, produced by a ^99^Mo/^99m^Tc generator eluent (Monrol, Istanbul, Turkey) with 0.9% saline, was supplied by the Department of Nuclear Medicine of Ege University. Both o-CIP and s-CIP were radiolabeled with ^99m^Tc using freshly prepared 50 μL of stannous (II) chloride (1 mg SnCI_2_.H_2_O/ 1 mL H_2_O) solution. After that, o-CIP and s-CIP were labeled with 1 mCi ^99m^TcO_4_ and mixed at 25 °C for 30 min. After incubation, radiolabeling efficiencies and radiofrequency (Rf) values were determined using thin-layer radio chromatography (TLRC). In radiochromatography studies, cellulose-coated sheets were used as the stationary phase and ethanol / ultrapure water / ammonium hydroxide (2:5:1) was used as the mobile phase. TLRC analysis was repeated 7 times to ensure the accuracy of the results.

Time-dependent behavior of ^99m^Tc-o-CIP and ^99m^Tc-s-CIP at room temperature was monitored using the TLRC method. Stability studies were performed at different time points (30, 60, 120, and 240 min) after radioiodination reaction.

Lipophilicity of any complex is specified by the logarithm of the distribution coefficient (logP) of the n-octanol and water. The log P values were then calculated by the log (CPS octanol phase/CPS phosphate buffer phase) formula (CPS: count per second).

### Antimicrobial tests and bacterial binding assay

#### Antimicrobial tests

Antimicrobial activities of cave bacteria substrate, o-CIP, and s-CIP were evaluated against 6 pathogenic microorganisms– 5 bacterial strains and a fungal strain: *Escherichia coli (E*. *coli)* (ATCC 9637), *Staphylococcus aureus (S*. *aureus)* (ATCC 29213), *Pseudomonas aeruginosa (P*. *aeruginosa)* (ATCC 39327), methicillin-resistant Staphylococcus aureus (*MRSA*) (ATCC MP-2), vancomycin-resistant enterococci (*VRE*) (ATCC MP-1), and Candida albicans (ATCC 10231)]. Antimicrobial activities were determined using the zone of inhibition test. For this, 0.1 ml bacterial suspensions, prepared using a McFarland 0.5 standard tube, were inoculated into Mueller Hinton Agar 20 medium, and spread with a Drigalski spatula. Discs impregnated with cave bacteria substrate, o-CIP, and s-CIP were placed in the inoculated Petri dishes and incubated at 20 °C for ≥ 24 hours. Zones of inhibition that formed at the end of the period were measured and the susceptibility of cave isolates to selected antibiotics was interpreted [9].

#### Bacterial binding assay

This experiment was performed in the presence of ^99m^Tc‐o-CIP and ^99m^Tc‐s-CIP under the minimum inhibitory concentration (MIC) values of antibiotics for *E*. *coli* (ATCC 9637), *S*. *aureus* (ATCC 29213), *P*. *aeruginosa* (ATCC 39327), *VRE* (ATCC MP-1), *SCCmec Type MRSA* (ATCC MP-2), and *Candida albicans* (ATCC 10231). Briefly, aliquots of suspensions of the harvested microorganisms containing ∼1.5 × 10^8^ colony-forming units (CFU) in 950 μL were prepared and added into microcentrifuge tubes. In addition, heat‐killed bacteria containing ∼1.5 × 10^8^ CFU in 950 μL were also prepared and added into microcentrifuge tubes as a control group. Then, ∼50 μL 0.37 MBq of radiolabeled antibiotics were added into microcentrifuge tubes. Then, radioactivity values in the tubes were counted to obtain initial activity values (A_0_, cps) with a Cd(Te) detector. The tubes were incubated at 37 °C for 30, 60, 120, and 240 min. At the end of the various incubation periods, the tubes were centrifuged at 5000 rpm for 10 min, the supernatants were removed, and the bacterial pellets were gently suspended in 1 mL of PBS (pH 7.4) and recentrifuged. Radioactivity values in the tubes were counted again with a Cd(Te) detector to obtain final radioactivity values (A_1_, cps). Binding percentage rates were calculated using the equation A_1_/A_0_ × 100. As a control group, 50 μL 0.37 MBq Nа^99m^ТсО_4_ was added into microcentrifuge tubes containing pathogenic microorganisms at 37 °C and incubated using the same incubation times. Bacterial binding assays were repeated 4 times to ensure the accuracy of the results.

### Statistical analysis

Statistical significance was assessed via one-way ANOVA and linear regression using GraphPad software. P values < 0.05 was considered statistically significant.

## Results

### LC-QTOF/MS and HPLC analysis

Ciprofloxacin is a widely used and widely studied antibiotic, given that it is a cost-effective antibiotic that is able to treat a variety of bacterial infections and can also be easily radiolabeled; however, due to its frequent use, it is limited by high rates of antibiotic resistance [10]. In our study, organic ciprofloxacin (o-CIP), produced by the *Micrococcus luteus* cave bacteria, was isolated. Table 1 shows the results from LC/Q-TOF/MS analysis of the *Micrococcus luteus* supernatant.

**Table 1:** Results from LC-QTOF/MS analysis of the *Micrococcus luteus* supernatant.

Compound Name	Compound Class	Formula	Score	Mass (m/z)
**Sucrose**	Sugar	C_12_H_22_O_11_	99.61	342.1162
**Phthalic acid Mono-2-ethylhexyl Ester**		C_16_H_22_O_4_	83.20	278.1524
**Mecetronium**		C_20_H_44_N	99.87	298.3476
**Levofloxacin**	Antibiotics	C_18_H_20_FN_3_O_4_	95.00	361.1434
**DL-Phenylalanine**		C_9_H_11_NO_2_	95.42	165.0783
**Clindamycin**	Antibiotics	C_18_H_33_C_l_N_2_O_5_S	97.54	424.1795
**Ciprofloxacin (o-CIP)**	Antibiotics	C_17_H_18_FN_3_O_3_	83.93	331.1323
**Betaine**		C_5_H_12_NO_2_	98.15	118.0868
**Benzododecinium (Ajatin)**		C_21_H_38_N	99.58	304.3004
**Arginine**	Amino acid	C_6_H_14_N_4_O_2_	97.07	174.1114

**Fig 2:**
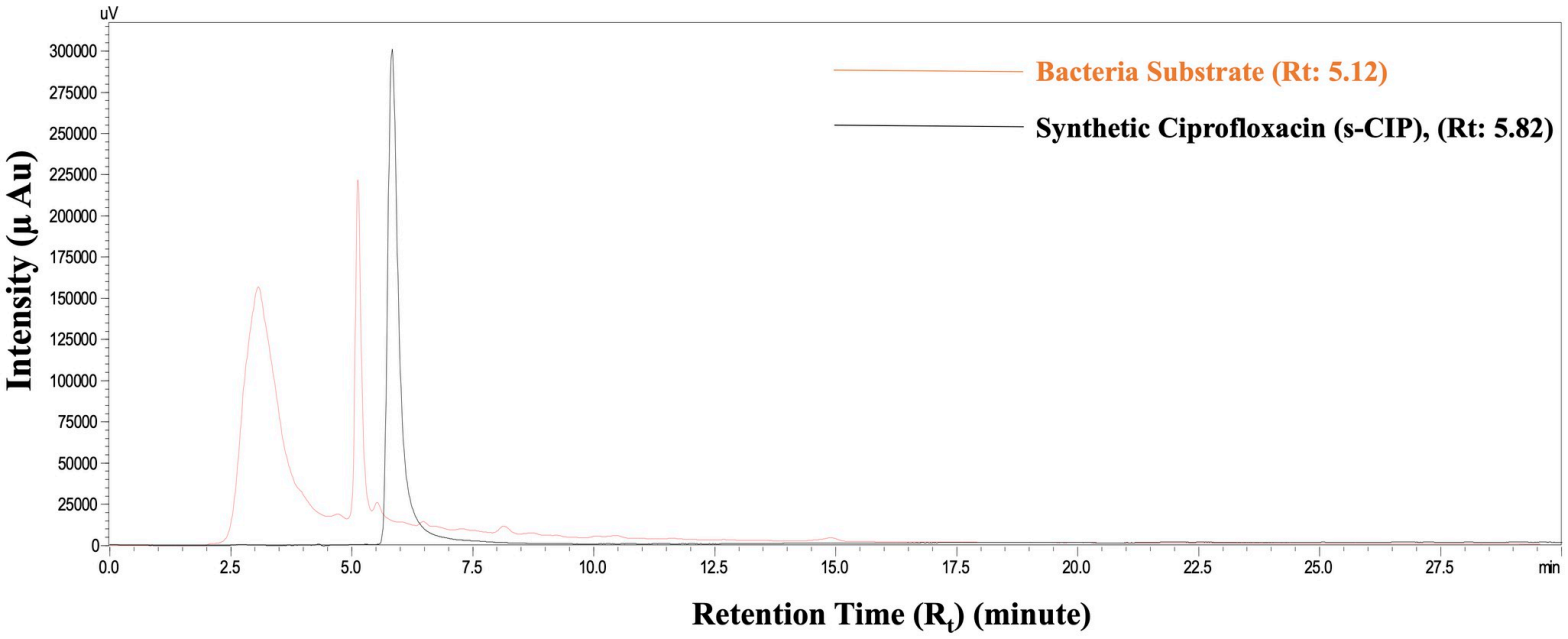
HPLC chromatogram of the bacteria substrate and s-CIP (n = 7).

As can be seen in Table 1, the bacteria produced 3 different organic antibiotics: levofloxacin, clindamycin, and ciprofloxacin. As ciprofloxacin is an antibiotic used in the treatment of many bacterial infections [11], o-CIP was purified from the cave bacteria nutrient medium using HPLC, and its synthetic counterpart s-CIP was also prepared as a standard for comparison (Fig 2).

As can be seen in Fig 2, the HPLC peak containing the bacterial substrate is close to the peak of s-CIP, with a retention time (Rt) of 5.12 compared with an Rt of 5.82 for s-CIP. This HPLC peak was defined as o-CIP. Based on the HPLC calibration curve of s-CIP, it was also quantified that 438 mg of o-CIP was purified using HPLC.

### Radiolabeling and quality control studies of o-CIP and s-CIP

**Fig 3:**
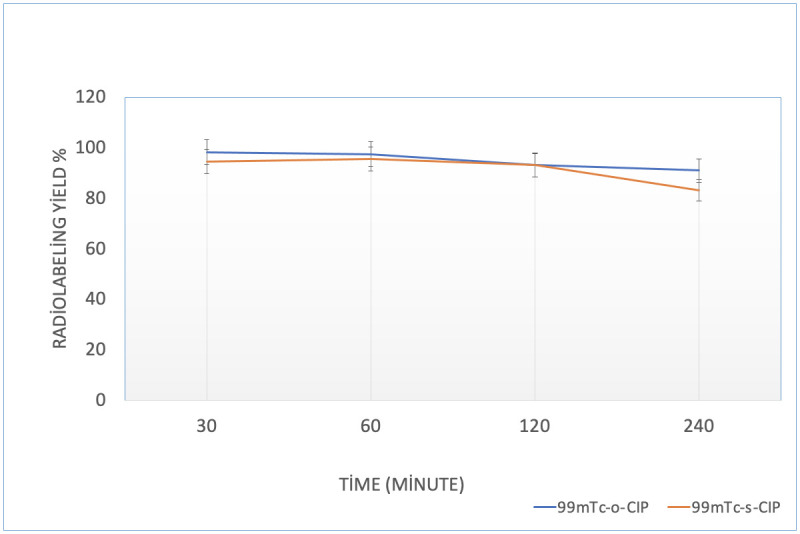
TLRC chromatogram of radiolabeled molecules.

The radiolabeling yield for o-CIP-^99m^Tc and s-CIP-^99m^Tc (n = 7) was calculated using TLRC. Rf values of ^99m^Tc, reduced ^99m^Tc, o-CIP-^99m^Tc, and s-CIP-^99m^Tc in developing media [n-butanol/bidistilled water/acetic acid (4:2:1)] were 0.87, 0.07, 0.82 and 0.83, respectively (Fig 3).

**Fig 4:**
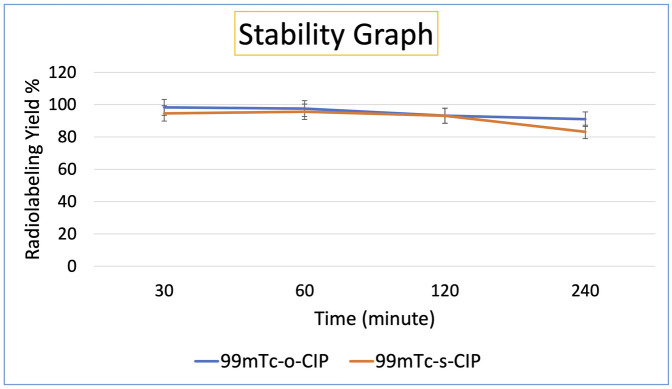
Stability graph of 99mTc-o-CIP and 99mTc-s-CIP.

Time-dependent changes of the radiochemical purity of ^99m^Tc-o-CIP and ^99m^Tc-s-CIP were monitored with stability studies at room temperature. ^99m^Tc-o-CIP maintained its stability with 97.60 ± 2.83% purity up to 24 h. ^99m^Tc-s-CIP demonstrated approximately 80% stability up to 24 h (Fig 4).

### Antimicrobial tests and bacterial binding assay

**Fig 5:**
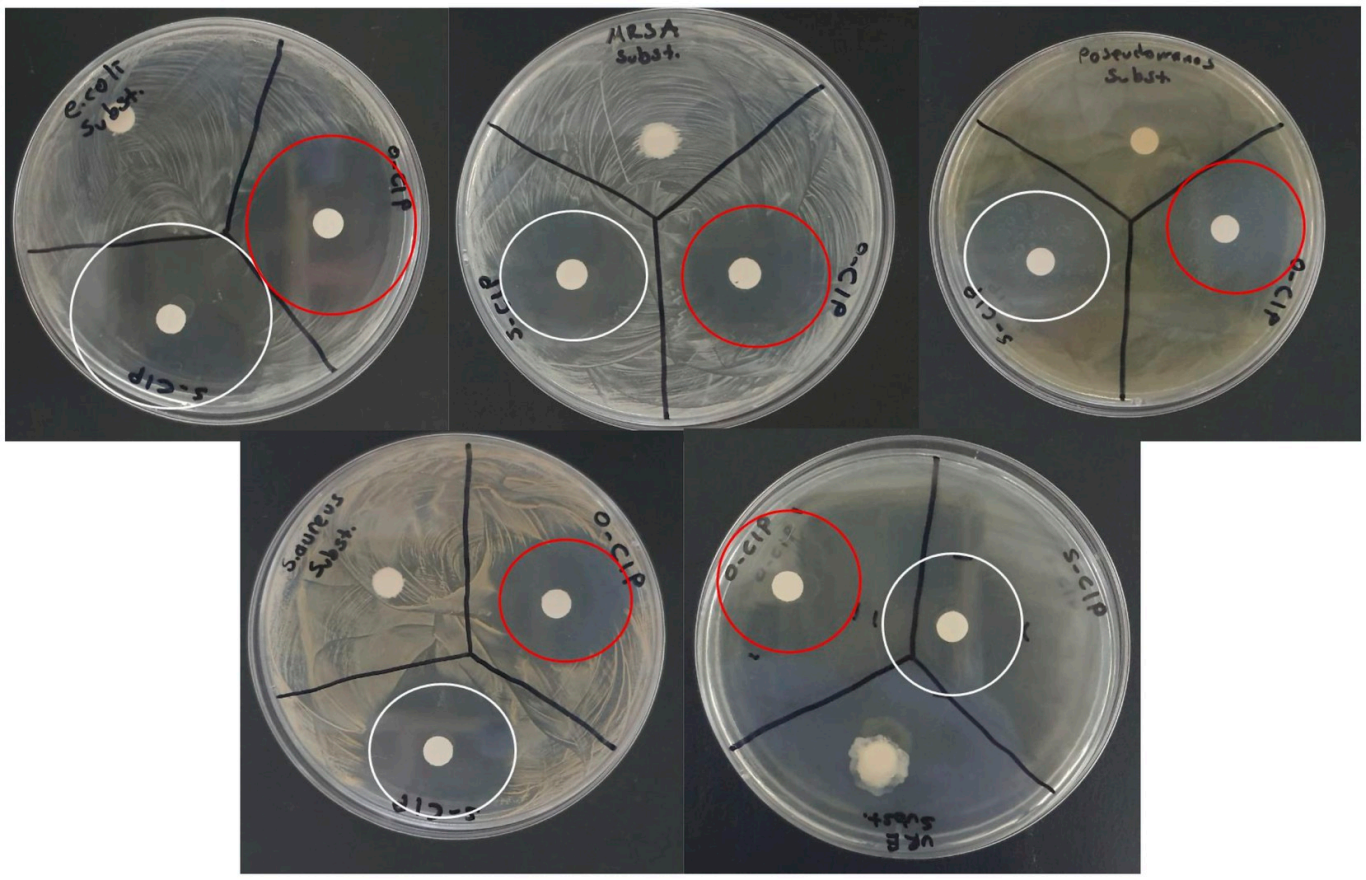
Zones of bacterial supernatant, o-CIP, and s-CIP, when added to *E*. *coli*, *MRSA*, *P*. *aeruginosa*, *S*. *aureus*, and *VRE*.

According to the zone of inhibition test, no zone formation was observed when bacteria supernatant was applied to the 6 selected pathogenic microorganisms. On the other hand, when o-CIP and s-CIP were applied, they formed zones with *P*. *aeruginosa*, *MRSA*, *VRE*, *E*. *coli*, and *S*. *aureus* (n = 4) (Fig 5). Based on the zone diameters measured using ImageJ software, o-CIP and s-CIP formed the same ratio of zones with *P*. *aeruginosa*, *VRE*, and *S*. *aureus*; s-CIP was 10 ± 1.12% (P ≤ 0.5) more effective than o-CIP with *E*. *coli*; and o-CIP was 5 ± 0.25% (P ≤ 0.5) more effective than s-CIP with *MRSA*.

**Fig 6:**
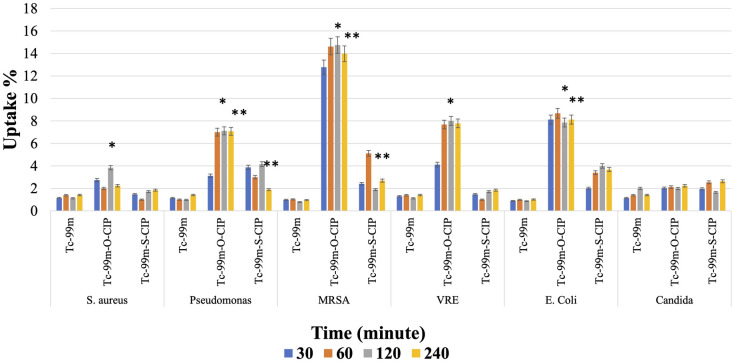
% Binding efficiency of ^99m^Tc, ^99m^Tc-o-CIP, and ^99m^Tc-s-CIP with pathogenic microorganisms.

The binding efficiency results of ^99m^Tc-o-CIP, ^99m^Tc-s-CIP and ^99m^Tc with the 6 pathogenic microorganisms are summarized in Fig 6. ^99m^Tc itself had a binding efficiency of 1.5% with all pathogenic microorganisms at the 240^th^ minute. Compared to ^99m^Tc-s-CIP, the binding efficiency of ^99m^Tc-o-CIP at the 240^th^ minute was approximately 4 times higher (7.08 ± 0.81 vs. 1.89 ± 0.49) with *P*. *aeruginosa*, approximately 5 times higher (13.98 ± 3.21% vs. 2.69 ± 0, 53) with *MRSA*, approximately 4 times higher (7.78 ± 0.81 vs. 1.84% ± 0.53) with *VRE*, and approximately 2 times higher (8.12 ± 1.01% vs. 3.68 ± 0.54) with *E*. *coli* (P ≤ 0.5 for all comparisons).

## Discussion

The discovery of organically produced bacteria dwelling within the depths of a remote cave has captivated the scientific community, offering unprecedented opportunities in the field of bacterial infection diagnostics. These remarkable microorganisms, uniquely adapted to their subterranean habitat, exhibit inherent characteristics that make them potential candidates for imaging-based detection methods. The integration of organically produced bacteria from cave environments and labeling with ^99m^Tc represents a novel approach in the field of bacterial infection diagnostics. This synergistic combination harnesses the inherent properties of the bacteria and the radiopharmaceutical capabilities of ^99m^Tc, leading to a promising avenue for non-invasive imaging-based detection of bacterial infections.

Antibiotics work in a number of ways; for example, some target ribosomes and nucleic acids, the building blocks of protein synthesis, thus hampering the construction and functioning of the bacterial cell wall and cell membrane [12]. Ciprofloxacin, a widely used antibiotic, works by inhibiting DNA replication [11]. While antibiotics can be highly effective in treating bacterial infection, the widespread use of antibiotics worldwide has led to the emergence of antibiotic resistant-bacteria [13]. For example, while almost all *S*. *aureus* strains were previously highly sensitive to ciprofloxacin, today this sensitivity has decreased due to the overuse of ciprofloxacin [14].

Ancient and intact cave ecosystems contain a unique microbial world, and to date, different caves around the world have been explored for potential bioactive compounds. A 2021 review by Zada et. al. highlighted that cave microorganisms may serve a potential source for new antimicrobial and anticancer drugs, addressing the threats of new pathogens and the rise of antibiotic resistance to existing pathogens [15]. Elsewhere, it has been suggested unexplored bacterial strains can produce novel antimicrobial compounds and that some bacteria have a higher potential to produce specific bioactive metabolites than other bacteria [16, 17].

In 2021, Paun et. al. obtained and characterized bacterial strains from the 13,000-year-old ice core of the Scarisoara ice cave in Romania, in an effort to take advantage of a habitat far away from human contamination and with extreme environmental conditions. They identified 68 bacterial isolates, with 17 strains representing putative new taxa, providing the first isolates and the first culture-based evidence of bacterial resistome and antimicrobial compound production in perennial ice deposited in caves from the late glacial period [18]. They also showed that some of the isolates inhibited the growth of clinically important Gram-positive and Gram-negative resistant strains and have promising metabolic properties against antibiotic resistance. Additionally, in 2021, a series of newly isolated strains of *Actinobacteria* from caves in the Tatra Mountains in Poland were studied [19]. One of the strains, *Streptomyces*, showed the ability to inhibit the growth of pathogenic bacteria and to reduce the viability of T47D cells. In addition, a variety of previously unknown (unclassified) molecules were detected using LC-MS. The researchers suggested that the *Streptomyces* strain isolated and characterized in their study could be used to synthesize a new bioactive compound with antibacterial, antifungal, and anticancer activities.

Most relevant to our study is a study by Rangseekaew et al. in 2019, which reported that karst caves in Turkey harbor actinobacteria, of which 62% of the isolates were found to be active against various microbial pathogens (Gram positive bacteria, Gram negative bacteria, yeast, and filamentous fungi) [20]. For this reason, we sought to conduct a further exploration of organically produced antibiotic from bacteria from existing cave ecosystems in Turkey. In our study, sediment was collected from an area that has survived in stable conditions for centuries within a cave environment far from human contamination. LC-QTOF/MS analysis determined that there were three antibiotics produced by the *Micrococcus luteus* cave bacterial strain, and of these three antibiotics, ciprofloxacin was selected and isolated using HPLC. It was decided to use ciprofloxacin because it is easy to obtain, convenient to purify, and frequently used in the clinical routine. The antimicrobial activity o-CIP was determined and compared to the antimicrobial activity of its commercial, synthetically produced counterpart.

In addition, both o-CIP and s-CIP were radiolabeled with ^99m^Tc, a widely used radionuclide in nuclear medicine. Of note, the differentiation of septic infection from sterile inflammation using ^99m^Tc-labeled ciprofloxacin was first performed in 1993, using a simple scintigraphy method. Since then, various studies have been conducted on the use of ^99m^Tc-labeled ciprofloxacin to differentiate bacterial from non-bacterial infections. For example, an *in vivo* infection model was created by Welling et al., in which radiolabeled ciprofloxacin was injected intravenously with ^99m^Tc and its binding efficiency with *S*. *aureus/MRSA* and *Candida* at 60 minutes was found to be 8 ± 0.1% and 2.6 ± 0.2%, respectively [21]. However, overall, results pertaining to the ability of ^99m^Tc-labeled ciprofloxacin to differentiate bacterial from non-bacterial infections have been inconclusive [22]. This may be because ciprofloxacin cannot be labeled directly with ^99m^Tc [23]. One method of preparing ^99m^Tc-labeled ciprofloxacin involves the addition of reducing agents to stabilize the +5 oxidation state of technetium (^99m^Tc Tricarbonyl Complex, ^99m^Tc(CO)_3_(LAN)) [24]. In our study, we used stannous (II) chloride for radiolabeling, resulting in a radiolabeling efficiency of 99.99% for ^99m^Tc-o-CIP and a radiolabeling efficiency of ^99m^Tc-s-CIP of 91.71%. In addition, in the stability experiments performed in our study, we found that ^99m^Tc-o-CIP has over 95% binding efficiency up to 240 minutes while ^99m^Tc-s-CIP was not stable up to 240 minutes. As such, we believe that ^99m^Tc-o-CIP is promising to be a more practical imaging agent than ^99m^Tc-s-CIP, allowing a longer timeframe for imaging at low concentrations.

In summary, our study shows that o-CIP, which we isolated from the *Micrococcus luteus* bacteria from the dark zone of Antalya’s "Yarık Sinkhole," can be developed as a new radiotracer especially for the imaging of infectious diseases based on radiolabeling efficiency results and antimicrobial test results when applied to pathogenic microorganisms. One limitation of our study is its preclinical nature, and thus it is necessary perform further studies beyond the preclinical setting to confirm the results. Along with further studies to determine the efficacy of o-CIP in inhibiting the growth of pathogenic bacteria and fungi, further studies should also be conducted to determine the efficacy of o-CIP in reducing the viability of cancer cells. It should also not be forgotten that there potentially other new antibiotics produced by cave bacteria that are waiting to be discovered, which will contribute further to progress in the field.

In conclusion, our study suggests that the use of organically produced antibiotics from cave bacteria is promising to yield a new generation of antibiotics to fight against bacterial infection, which will have a positive effect on infection-related morbidity and mortality rates, thus paving the way for a healthier world.
